# Neural Network Copulas for Generating Synthetic Test Data Preserving Psychometric Properties

**DOI:** 10.3390/jintelligence14050077

**Published:** 2026-05-02

**Authors:** Juyoung Jung, Minho Lee, Won-Chan Lee

**Affiliations:** 1 Educational Measurement and Statistics, Psychological and Quantitative Foundations, University of Iowa, Iowa City, IA 52242, USA; juyoung-jung@uiowa.edu; 2 Department of Psychology, University of Notre Dame, Notre Dame, IN 46556, USA; mlee57@nd.edu

**Keywords:** synthetic data generation, neural network copula, item response theory, intelligence assessment, psychometric properties, privacy protection

## Abstract

In intelligence research, the sharing of item response data from cognitive ability assessments is often restricted by privacy concerns, while traditional parametric simulation methods frequently fail to capture complex response dependencies. This study proposes a neural network copula (NNC) framework for generating synthetic dichotomous item response data that preserves essential psychometric properties without revealing sensitive examinee information. By decoupling the modeling of marginal item probabilities from the dependence structure using a deep autoencoder and kernel density estimation, the framework accommodates the discrete nature of binary item response data while minimizing distributional assumptions. Validation against large-scale empirical data demonstrated high correspondence across multiple facets. At the data consistency level, the NNC-based synthetic data reproduced total score distributions and inter-item correlations. Psychometrically, the method yielded consistent item characteristic curve parameter estimates, item fit statistics, and test information functions. Furthermore, Monte Carlo replications demonstrated algorithmic stability and inferential precision.

## 1. Introduction

Synthetic data have been adopted in quantitative disciplines as a mechanism for privacy protection, reproducible research, and methodological benchmarking (Drechsler, 2011; Reiter, 2005). The core principle underlying synthetic data generation is to replace observed records with simulated observations drawn from models estimated on the original data, thereby breaking the direct linkage to real individuals while preserving inferential utility, the degree to which statistical inferences drawn from synthetic data replicate those obtainable from the original (Raab et al., 2016; Raghunathan et al., 2003; Rubin, 1993). Traditional approaches to generating synthetic item response data have relied primarily on parametric item response theory (IRT) simulation, wherein item parameters are estimated from empirical data and new responses are generated by sampling abilities from assumed distributions (Baker & Kim, 2004; Harwell, 1997). While conceptually straightforward, this paradigm faces important limitations. Standard IRT models impose rigid assumptions regarding the functional form of item characteristic curves (ICCs), typically relying on logistic or normal ogive functions (Hambleton et al., 1991). These parametric specifications may inadequately represent the complex response processes observed in real assessments, leading to model misspecification and synthetic data that diverge from original distributional features (Junker & Sijtsma, 2001; Sijtsma & Molenaar, 2002). 

Standard IRT models traditionally assume strict unidimensionality and conditional independence. While multidimensional IRT theoretically accommodates multiple traits, it requires pre-specifying the number of latent dimensions (Reckase, 1985). Exploratory factor analysis of categorical data (Bartholomew, 1980; Y. Yang & Xia, 2015) and multiple MIRT parameterizations, including compensatory and non-compensatory forms (Wang & Nydick, 2015), offer some flexibility. However, these approaches limit analysis by decomposing item dependencies into a finite set of latent factors. Any residual inter-item covariance falling outside this specified structure is discarded rather than modeled. When data exhibit complex local item dependence (Chen & Thissen, 1997; Yen, 1984), this oversimplified latent representation distorts underlying correlation patterns and violates the local independence assumption (Stout, 1987). Beyond these structural constraints, alternative methods like parametric bootstrapping (Efron & Tibshirani, 1994), multiple imputation (Rubin, 2018), and latent class modeling (Formann, 2007; Vermunt, 2004) struggle to balance flexibility with scalability. Relying on strict distributional assumptions, these approaches fail to capture the complex nonlinear dependencies inherent in large-scale binary response matrices (Chalmers, 2016; Si & Reiter, 2013).

Recent advances in deep learning have introduced flexible, data-driven alternatives for synthetic data generation that relax the rigid assumptions of parametric simulation (LeCun et al., 2015). Deep generative models, including generative adversarial networks (GANs) (I. J. Goodfellow et al., 2014) and variational autoencoders (VAEs) (Kingma & Welling, 2014; D. J. Rezende et al., 2014), have demonstrated the capacity to learn complex, nonlinear dependencies without explicit parametric specification (Xu et al., 2019). However, directly applying these methods to item response data while preserving psychometric properties remains challenging. GANs are often unstable to train and susceptible to mode collapse, resulting in incomplete representation of response pattern variability (Arjovsky et al., 2017; Salimans et al., 2016). Similarly, VAEs typically impose strong distributional assumptions on the latent space that may not align with ability distributions or psychometric constructs (Bowman et al., 2016). In both cases, the absence of an explicit mechanism for preserving marginal item properties means that downstream IRT-based analyses may be compromised, a limitation the present framework directly addresses.

Copula-based modeling, grounded in Sklar’s theorem (Sklar, 1959), offers a complementary framework by decomposing multivariate distributions into univariate marginals and an explicit dependence structure (Joe, 2014; Nelsen, 2006). This separation is particularly well suited to psychometric data; marginal response probabilities can be directly estimated from observed item frequencies, while the copula captures complex dependencies induced by latent traits (Braeken et al., 2007; Nikoloulopoulos & Joe, 2015). Traditional copula approaches in psychometrics have employed parametric copula families such as Gaussian or factor copulas (Kadhem & Nikoloulopoulos, 2023a, 2023b). Recent developments show that neural networks can automate copula construction, substantially improving distributional flexibility without requiring manual specification of parametric copula families (Letizia et al., 2025; R.-S. Yang et al., 2024). As a result, neural network copula (NNC) models can provide a principled mechanism for generating synthetic item response data that preserve both marginal item properties and dependence structures essential for IRT-based analyses.

In this study, we propose an NNC model for generating synthetic dichotomous item response data in educational and psychological assessments. The approach follows a three-stage process: (1) transforming binary responses to pseudo-uniform marginals via randomized probability integral transforms (Genest et al., 2009; Rüschendorf, 2009), (2) learning dependence structures using deep autoencoders (Kingma & Welling, 2014; D. J. Rezende et al., 2014), and (3) generating synthetic data via inverse transformations. By injecting controlled stochasticity, the pseudo-uniform transform resolves the tie-breaking problem inherent in discrete marginals (Genest et al., 2009; Mendes et al., 2007). This ensures that the variates are marginally uniform, fulfilling a prerequisite for Sklar’s theorem (Nelsen, 2006; Sklar, 1959) while preserving inter-item dependence. Critically, item discrimination and difficulty are maintained through the empirical marginal probabilities in the final binary generation step, allowing the design to accommodate the latent variable structure while minimizing distributional assumptions. Finally, to address the inherent risk of distributional divergence and the creation of plausible but statistically unfaithful records (Xu et al., 2019), we utilize a multi-faceted validation framework.

Despite increasing interest in neural synthetic data generation (Choi et al., 2017; Xu et al., 2019), current validation practices often focus on distributional similarity rather than meaningful psychometric criteria (Drechsler & Reiter, 2011). In particular, the reproduction of sampling variability relevant for statistical inference has received little systematic attention (Raab et al., 2016). To address this gap, we conduct an IRT-grounded psychometric validation (Baker & Kim, 2004; Hambleton et al., 1991) evaluating data-level consistency, parameter recovery, and item and test information accuracy. Crucially, we extend standard protocols by comparing empirical asymptotic standard errors with Monte Carlo standard errors (MCSEs) (Robert & Casella, 2004) derived from repeated synthetic generation. This comparative analysis assesses whether the NNC framework preserves both point estimates and the original inferential uncertainty. Through an empirical application to a large-scale numerical reasoning assessment of fluid intelligence, we demonstrate that the proposed approach accurately reproduces essential psychometric properties while protecting examinee anonymity. Generating such structurally faithful synthetic data is critical for intelligence research; operational datasets are rarely shareable due to privacy protocols, though they are essential for developing assessments and benchmarking cognitive measurement models.

## 2. Method

### 2.1. Item Response Theory Framework

#### 2.1.1. Two-Parameter Logistic Model

The two-parameter logistic (2PL) model (Birnbaum, 1968) served as the psychometric framework for parameter estimation and validation of psychometric properties. The present framework is scoped to dichotomous (binary) response data. Under the 2PL model, let $Y_{ik}$ denote the observed binary response of examinee *i* (i=1,…,n) to item *k* (k=1,…,K), where $Y_{ik}=1$ indicates a correct response and $Y_{ik}=0$ indicates an incorrect response. The probability of a correct response for an examinee with latent ability $\theta_{i}$, given item discrimination $a_{k}$ and difficulty $b_{k}$, is specified as:

(1) $$P\left(Y_{ik}=1|\theta_{i},a_{k},b_{k}\right)=\frac{1}{1+\exp[-a_{k}\left(\theta_{i}-b_{k}\right)]},$$

where $\theta_{i}$ represents the latent ability, $a_{k}>0$ denotes the discrimination parameter indicating how effectively item *k* differentiates among ability levels, and $b_{k}$ represents the difficulty parameter corresponding to the ability level at which the probability of a correct response equals 0.5. The 2PL model relies on three core assumptions: unidimensionality (responses are governed by a single latent trait), local independence (responses are conditionally independent given $\theta_{i}$), and monotonicity (higher ability increases the probability of success) (Embretson & Yang, 2012; Lord et al., 1968).

#### 2.1.2. Parameter Estimation

IRT parameters were estimated using marginal maximum likelihood (MML) with an expectation-maximization (EM) algorithm (Bock & Aitkin, 1981). The marginal likelihood integrates over the latent ability distribution, defined as:

(2) $$L\left(a,b|Y\right)=\prod_{i=1}^{n}\int \left[\prod_{k=1}^{K}P\left(Y_{ik}|\theta ,a_{k},b_{k}\right)\right]\phi \left(\theta \right)\;d\theta ,$$

where ϕ(θ) denotes the standard normal density representing the population ability distribution. The EM algorithm alternates between computing expected sufficient statistics based on posterior ability distributions (E-step) and updating item parameters to maximize the expected complete-data log-likelihood (M-step) (Baker & Kim, 2004; Bock & Aitkin, 1981).

Following item parameter estimation, individual ability parameters were obtained via Expected A Posteriori (EAP) estimation (Bock & Mislevy, 1982). The EAP method estimates the latent trait ${\hat{\theta }}_{i}$ as the expected value of the posterior distribution of ability, conditional on the observed response pattern $y_{i}$ and the estimated item parameters:

(3) $${\hat{\theta }}_{i,\mathrm{EAP}}=\frac{\int_{-\infty }^{\infty }\theta L\left(y_{i}|\theta \right)\phi \left(\theta \right)\;d\theta }{\int_{-\infty }^{\infty }L\left(y_{i}|\theta \right)\phi \left(\theta \right)\;d\theta },$$

where $L(y_{i}|\theta )$ represents the likelihood function and ϕ(θ) is the standard normal prior. The integrals are approximated using the Gauss–Hermite quadrature (Thissen et al., 1995). EAP estimation was selected because it minimizes mean squared error over the population and yields stable, finite estimates even for extreme response patterns, for which maximum likelihood estimates are undefined (Bock & Mislevy, 1982; Thissen et al., 2001).

### 2.2. Neural Network Copula Methodology

This section outlines the proposed NNC framework for modeling binary item response data. The complete algorithmic procedures for model training and synthetic data generation are detailed in Appendix A.

#### 2.2.1. Copula Theoretical Foundation

Copula theory provides the mathematical foundation for our approach by enabling the separation of marginal distributions from dependence structures (Nelsen, 2006; Sklar, 1959). Sklar’s theorem establishes that any multivariate distribution function *F* can be uniquely decomposed as:

(4) $$F\left(y_{1},\ldots ,y_{K}\right)=C\left(F_{1}\left(y_{1}\right),\ldots ,F_{K}\left(y_{K}\right)\right),$$

where $F_{k}$ are univariate marginal cumulative distribution functions and $C:{\left[0,1\right]}^{K}\to \left[0,1\right]$ is the copula function capturing the dependence structure (Sklar, 1959). This decomposition is particularly valuable for psychometric data: marginal response probabilities $P(Y_{k}=1)$ can be directly estimated from observed frequencies, while the copula *C* encodes complex dependencies induced by the underlying latent structure without requiring explicit specification of the latent variable model (Braeken et al., 2007; Fox & Marianti, 2016; Nikoloulopoulos & Joe, 2015).

For discrete binary data, we employ a pseudo-copula approach via the randomized probability integral transform (Genest et al., 2009; Rüschendorf, 2009). Given binary responses $Y_{ik}\in \left\{0,1\right\}$ with marginal probability $p_{k}=P\left(Y_{ik}=1\right)$, we construct pseudo-uniform variates $U_{ik}$ as:

(5) $$U_{ik}=\left\{\begin{array}{cc} \mathrm{Uniform}(1-p_{k},\;1) & \mathrm{if}\;Y_{ik}=1,\\ \mathrm{Uniform}(0,\;1-p_{k}) & \mathrm{if}\;Y_{ik}=0. \end{array}\right.$$

This stochastic transformation ensures that marginally $U_{ik}∼\mathrm{Uniform}\left(0,1\right)$ while preserving the underlying dependence structure, yielding a pseudo-uniform matrix $U=\left[U_{ik}\right]\in {\left[0,1\right]}^{n\times K}$ suitable for copula modeling (Genest et al., 2009).

It is important to clarify what this transformation does and does not do, as the role of the pseudo-uniform step within the broader pipeline can be misread. Equation (5) is a standard preprocessing technique required by Sklar’s theorem when the response variables are discrete. Because discrete distributions have no unique copula representation without a continuity correction, the randomized probability integral transform resolves the tie-breaking problem by injecting controlled stochasticity, ensuring that the transformed variates are marginally uniform (Genest et al., 2009; Mendes et al., 2007; Rüschendorf, 2009). Crucially, this step does *not* replace the IRT model, nor does it discard information about item discrimination (ICC steepness) or difficulty. Those psychometric quantities are preserved through the empirical marginal probabilities ${\hat{p}}_{k}$, which govern the final binary generation step in Equation (12). Unlike deterministic transformations that can introduce artifacts at boundaries, this randomized approach maintains distributional properties essential for accurate dependence modeling (Rüschendorf, 2009).

#### 2.2.2. Deep Autoencoder Architecture

The NNC employs an encoder–decoder architecture designed to isolate and learn the latent dependence structure underlying the item responses. Functionally, the encoder serves as a non-linear feature extraction mechanism, mapping the observed pseudo-uniform variates to a latent space via:

(6) $$z_{i}=f_{\mathrm{enc}}\left(u_{i};\;\Theta_{\mathrm{enc}}\right),$$

where $u_{i}$ is the *i*-th row of the pseudo-uniform matrix U, and $z_{i}$ represents the latent code. By compressing the input, the encoder forces the model to capture the essential covariance structure of the data rather than simply memorizing individual response patterns.

Conversely, the decoder acts as the generative component, reconstructing the uniform marginals from these latent codes:

(7) $${\hat{u}}_{i}=f_{\mathrm{dec}}\left(z_{i};\;\Theta_{\mathrm{dec}}\right),$$

where $\Theta_{\mathrm{dec}}$ denotes the decoder parameters. This reconstruction process ensures that the latent codes $z_{i}$ retain sufficient information to recover the original multivariate distribution.

Our implementation employs a multi-layer feedforward architecture to approximate these complex mappings (Hinton & Salakhutdinov, 2006). The encoder comprises three hidden layers with dimensions [256, 128, 64]. This decreasing architecture creates a hierarchical compression funnel. By initializing with a high-capacity layer, the network can capture broad, non-linear associations among items, while the subsequent progressive reduction forces the model to distill these interactions into increasingly abstract representations, thereby minimizing the information loss often associated with abrupt dimensionality reduction (I. Goodfellow et al., 2016).

Each layer utilizes rectified linear unit (ReLU) activation, σ(x)=max(0,x) (Glorot et al., 2011; I. Goodfellow et al., 2016), to model non-linear interactions between items. To ensure convergence and generalization, we apply batch normalization (Ioffe & Szegedy, 2015) to stabilize internal covariate shifts and dropout regularization (rate 0.2) (Srivastava et al., 2014) to prevent co-adaptation of neurons. The final encoder layer projects to *d* latent dimensions without activation, allowing the latent space to span the full real number line. The specific value of this dimensionality parameter, *d*, is determined via sensitivity analysis to balance reconstruction fidelity, dependence preservation, and model parsimony (see Section 4.1). The decoder mirrors this architecture in reverse but concludes with a sigmoid output activation, $\sigma \left(x\right)={\left(1+e^{-x}\right)}^{-1}$. This final activation is critical for the copula framework, as it strictly constrains the reconstructed outputs ${\hat{u}}_{i}$ to the unit interval [0,1], preserving the probabilistic interpretation of the uniform marginals required by Sklar’s theorem. This sigmoid constraint is a standard architectural choice for autoencoder outputs bounded to [0,1] (I. Goodfellow et al., 2016) and is functionally distinct from the logistic ICC in Equation (1), which models item response probabilities as a function of ability.

#### 2.2.3. Training Objective

The model optimizes a composite loss function designed to simultaneously learn the dependence structure while strictly enforcing the distributional constraints required by Sklar’s theorem:

(8) $$L\left(\Theta \right)=L_{\mathrm{recon}}\left(\Theta \right)+\lambda \;L_{\mathrm{marginal}}\left(\Theta \right).$$

The primary component, reconstruction loss ($L_{\mathrm{recon}}$), quantifies the autoencoder’s ability to compress and recover the multivariate dependence structure. We minimize the mean squared error between the input pseudo-uniform variates and the reconstructed outputs:

(9) $$L_{\mathrm{recon}}\left(\Theta \right)=\frac{1}{nK}\sum_{i=1}^{n}\sum_{k=1}^{K}{\left(U_{ik}-{\hat{U}}_{ik}\right)}^{2}.$$

By minimizing this error, the model is forced to encode the essential inter-item correlations into the latent space z.

The secondary component, marginal preservation loss ($L_{\mathrm{marginal}}$), acts as a regularizer to prevent “marginal collapse,” where the model might drift away from the required uniform distribution to minimize reconstruction error. This term penalizes deviations of the reconstructed column means from the theoretical expectation of a Uniform(0,1) distribution (which is 0.5):

(10) $$L_{\mathrm{marginal}}\left(\Theta \right)=\frac{1}{K}\sum_{k=1}^{K}{\left[\frac{1}{n}\sum_{i=1}^{n}{\hat{U}}_{ik}-0.5\right]}^{2}.$$

Ensuring that $E[{\hat{U}}_{\cdot k}]\approx 0.5$ anchors the generator to the correct marginal domain, which is critical for the subsequent inverse probability integral transform. The weighting parameter λ=0.1 was empirically selected to balance these objectives. It provides sufficient regularization to maintain distributional properties without overpowering the gradient signal needed to learn complex dependencies (I. Goodfellow et al., 2016). Model training employed the Adam optimizer (Kingma & Ba, 2015) with an initial learning rate η=0.001 and a mini-batch size of 64. To ensure convergence to a stable minimum, learning rate scheduling via “ReduceLROnPlateau” reduced the rate by 50% when validation loss plateaued for 10 consecutive epochs. Training continued until validation loss failed to improve for 20 consecutive epochs (early stopping), ensuring the retention of the generalizable model configuration (Prechelt, 1998).

#### 2.2.4. Synthetic Data Generation

Synthetic data generation proceeds through a four-stage pipeline designed to operationalize the learned copula model while preserving the complex distributional features of the latent space. First, latent representations extracted via the encoder are modeled using non-parametric kernel density estimation (KDE). This choice addresses the shrinking phenomenon often observed in deterministic autoencoder latent spaces, where parametric (e.g., Gaussian) assumptions can underestimate the density of extreme scores (Schmidt & Stadtmüller, 2006). The empirical distribution of latent codes (z) is estimated as:

(11) $$\hat{f}\left(z\right)=\frac{1}{nh^{d}}\sum_{i=1}^{n}K\;\left(\frac{z-z_{i}}{h}\right),$$

where $z_{i}$ are the training data latent vectors, K(·) is the standard Gaussian kernel, and *d* is the dimensionality of the latent space.

The bandwidth parameter *h* controls the smoothness of the estimated density and directly affects tail preservation in the synthetic data. Although *h* can be optimized via cross-validation to maximize held-out log-likelihood (Scott, 2015), in practice it is selected to balance the tradeoff between bias and variance in the latent distribution. In the present study, *h* is determined through a structured sensitivity analysis (see Section 4.1), evaluating its impact on dependence preservation and psychometric recovery. Specifically, the latent codes exhibited moderate variance with meaningful tail density corresponding to high- and low-ability examinees. A bandwidth in the range of *h* = 0.2 to 0.3, relative to the data-adaptive Silverman rule-of-thumb estimate of h≈[Z], mitigates over-smoothing and ensures better preservation of extreme scores in the synthetic output, which is critical for accurately capturing extreme ability levels in cognitive assessment contexts (Silverman, 1986).

Following distribution characterization, synthetic generation proceeds by sampling new latent codes $z^{\mathrm{syn}}$ directly from the estimated density $\hat{f}\left(z\right)$. These sampled codes are mapped back to the pseudo-uniform space via the decoder, where the sigmoid activation ensures values remain within [0,1]. To recover the discrete binary format, these continuous probabilities are subjected to an inverse probability integral transform:

(12) $$Y_{ik}^{\mathrm{syn}}=I\;\left\{U_{ik}^{\mathrm{syn}}>1-{\hat{p}}_{k}\right\},$$

where I{·} denotes the indicator function and ${\hat{p}}_{k}$ represents the empirical marginal probability observed in the training data. This thresholding mechanism guarantees that $E\left[Y_{ik}^{\mathrm{syn}}\right]={\hat{p}}_{k}$, thereby preserving item difficulty by construction (Genest et al., 2009). Crucially, this pipeline effectively decouples the modeling of complex dependence structures (managed by the neural network and KDE) from the preservation of marginal properties, leveraging the core advantage of the copula framework to ensure psychometric validity (Nelsen, 2006; Sklar, 1959).

### 2.3. Validation Metrics and Criteria

To evaluate the utility of the proposed NNC for synthetic item response data, we employed a multi-faceted validation framework following established IRT validation practice (Baker & Kim, 2004; Hambleton et al., 1991). The framework is organized into four complementary facets, specifically data-level consistency, psychometric properties, statistical inference precision, and algorithmic stability.

#### 2.3.1. Data-Level Consistency

The evaluation of data-level consistency focused on whether the synthetic data preserved statistical features of the original response matrix. First, generative quality at the respondent level was validated by comparing observed total score distributions. Second, univariate difficulty hierarchies were evaluated by contrasting the proportion of correct responses (marginal probability) for each item in the real data (${\hat{p}}_{k}^{\mathrm{real}}$) against the synthetic data (${\hat{p}}_{k}^{\mathrm{syn}}$). Linear agreement was assessed via Pearson correlation (*r*), and the magnitude of deviation was quantified using the mean absolute deviation (MAD):

(13) $${\mathrm{MAD}}_{\mathrm{marginal}}=\frac{1}{K}\sum_{k=1}^{K}\left|{\hat{p}}_{k}^{\mathrm{real}}-{\hat{p}}_{k}^{\mathrm{syn}}\right|.$$

MAD was preferred over RMSD for marginal probabilities because it provides an interpretable average absolute discrepancy on the original probability scale, without disproportionate influence from items with large individual deviations; RMSD is reported for IRT parameters, where sensitivity to outlying items is more diagnostically relevant.

Third, dependencies were assessed by computing pairwise inter-item correlation matrices for both datasets. The discrepancy between the matrices was quantified using the root mean square residual (RMSR):

(14) $$\mathrm{RMSR}=\sqrt{\frac{2}{K(K-1)}\sum_{k<l}{\left(r_{kl}^{\mathrm{real}}-r_{kl}^{\mathrm{syn}}\right)}^{2}},$$

where $r_{kl}$ represents the Pearson correlation between items *k* and *l*.

#### 2.3.2. Psychometric Properties

This dimension assesses whether the synthetic data maintains the structural and measurement characteristics essential for IRT-based inference. First, the consistency between IRT parameters estimated from synthetic data (${\hat{\psi }}^{\mathrm{syn}}$) and empirical data (${\hat{\psi }}^{\mathrm{real}}$) was evaluated. Rank-order preservation was quantified using Pearson correlation coefficients (*r*). The magnitude of parameter discrepancy was evaluated using the MAD and the root mean squared deviation (RMSD):

(15) $$\begin{array}{cc} {\mathrm{MAD}}_{\psi } & =\frac{1}{K}\sum_{k=1}^{K}\left|{\hat{\psi }}_{k}^{\mathrm{real}}-{\hat{\psi }}_{k}^{\mathrm{syn}}\right|, \end{array}$$

(16) $$\begin{array}{cc} {\mathrm{RMSD}}_{\psi } & =\sqrt{\frac{1}{K}\sum_{k=1}^{K}{\left({\hat{\psi }}_{k}^{\mathrm{real}}-{\hat{\psi }}_{k}^{\mathrm{syn}}\right)}^{2}}. \end{array}$$

These metrics were computed separately for discrimination (*a*) and difficulty (*b*) parameters. Additionally, the consistency of the latent trait distributions was evaluated by comparing kernel density estimates of the EAP ability scores (θ) derived from both datasets.

Second, to assess precision across the latent trait continuum, item information functions (IIF), test information functions (TIF), and conditional standard errors of measurement (CSEM) were computed over a uniform grid of 100 points, $\theta_{q}\in \left[-4,4\right]$. For the 2PL model, the information contributed by item *k* is defined as:

(17) $$I_{k}\left(\theta \right)=a_{k}^{2}\;P_{k}\left(\theta \right)\left[1-P_{k}\left(\theta \right)\right],$$

where $P_{k}\left(\theta \right)$ is the item response function. The term $P_{k}\left(\theta \right)\left[1-P_{k}\left(\theta \right)\right]$ is the Bernoulli variance of the item response, reflecting maximum information where response probability is most uncertain (i.e., near $\theta =b_{k}$). The aggregate TIF was calculated as $I\left(\theta \right)=\sum_{k=1}^{K}I_{k}\left(\theta \right)$. Consistency was quantified by calculating the Pearson correlation (*r*) between the real and synthetic TIF vectors, as well as between the CSEM vectors, across the evaluation grid.

Third, to verify probabilistic plausibility of the generated response patterns, Infit and Outfit mean square (MNSQ) statistics were computed based on standardized residuals $z_{ik}=\left(Y_{ik}-P_{ik}\right)/\sqrt{P_{ik}\left(1-P_{ik}\right)}$ (Hambleton et al., 1991; Wright & Stone, 1979). The Infit statistic weights residuals by their variance, making it sensitive to unexpected response patterns when the examinee’s ability is close to the item difficulty, while the Outfit statistic is an unweighted mean square sensitive to unexpected responses far from an examinee’s ability level:

(18) $$\begin{array}{cc} {\mathrm{Infit}}_{k} & =\frac{\sum_{i=1}^{n}W_{ik}\;z_{ik}^{2}}{\sum_{i=1}^{n}W_{ik}}, \end{array}$$

(19) $$\begin{array}{cc} {\mathrm{Outfit}}_{k} & =\frac{1}{n}\sum_{i=1}^{n}z_{ik}^{2}, \end{array}$$

where $W_{ik}=P_{ik}\left(1-P_{ik}\right)$ represents the Bernoulli variance of the binary response. Values within the acceptable range [0.7, 1.3] indicate adequate fit (Wright & Stone, 1979), while values below 0.5 indicate severe under-dispersion. The correspondence between the fit profiles of the real and synthetic data was assessed to ensure the generative model did not introduce aberrant response patterns (over-dispersion) or artificially deterministic dependencies (under-dispersion).

#### 2.3.3. Statistical Inference Precision

To assess whether the synthetic data appropriately reproduced sampling variability, we conducted a Monte Carlo analysis with M=500 independent synthetic data generations. The MCSE for each parameter $\psi_{k}$ was computed as:

(20) $${\mathrm{SE}}_{\mathrm{mc}}\left(\psi_{k}\right)=\sqrt{\frac{1}{M-1}\sum_{m=1}^{M}\;{\left({\hat{\psi }}_{k}^{\left(m\right)}-{\bar{\psi }}_{k}\right)}^{\;2}}.$$

Inferential validity was evaluated by comparing these MCSEs against the asymptotic standard errors (ASE) derived from the real data. The ASE for the item parameter vector $\psi_{k}={\left[a_{k},\;b_{k}\right]}^{⊤}$ is calculated as the square root of the diagonal elements of the inverse Fisher information matrix $I(\psi_{k})$ (Baker & Kim, 2004):

(21) $${\mathrm{SE}}_{\mathrm{asym}}\left(\psi_{k}\right)=\sqrt{\mathrm{diag}\;\left(I{\left(\psi_{k}\right)}^{-1}\right)}.$$

The ratio ${\mathrm{SE}}_{\mathrm{mc}}/{\mathrm{SE}}_{\mathrm{asym}}$ serves as the primary inferential validity index, with values near 1.0 indicating that the synthetic data reproduces the precision of the real data, values above 1.0 indicating conservative standard errors, and values below 1.0 indicating over-smoothing of sampling variability. It is important to note that model-based ASEs often underestimate uncertainty in empirical data due to inevitable model misspecification. Consequently, discrepancies where ${\mathrm{SE}}_{\mathrm{mc}}>{\mathrm{SE}}_{\mathrm{asym}}$ may reflect the synthetic data capturing full generative variability rather than a deficiency in precision.

#### 2.3.4. Algorithmic Stability

The preceding three facets assess the fidelity of synthetic data produced by a single trained model. Algorithmic stability addresses a distinct and complementary question: whether the results are reproducible across different random initializations of the neural network. This confirms that the observed fidelity reflects a stable structural representation of the data rather than a fortunate outcome of a particular optimization trajectory. To quantify stability, the complete training and generation pipeline is repeated across multiple independent execution runs, each utilizing a distinct random seed. For every iteration, the NNC is retrained from scratch to generate a new synthetic dataset. By evaluating the variance of the primary validation metrics across these independent runs, we can determine the robustness of the model. A low variance across executions indicates that the NNC converges to an equivalent generative solution regardless of initialization, satisfying the reproducibility requirement for practical deployment.

## 3. Analysis

### 3.1. Analytical Framework

**Figure 1:**
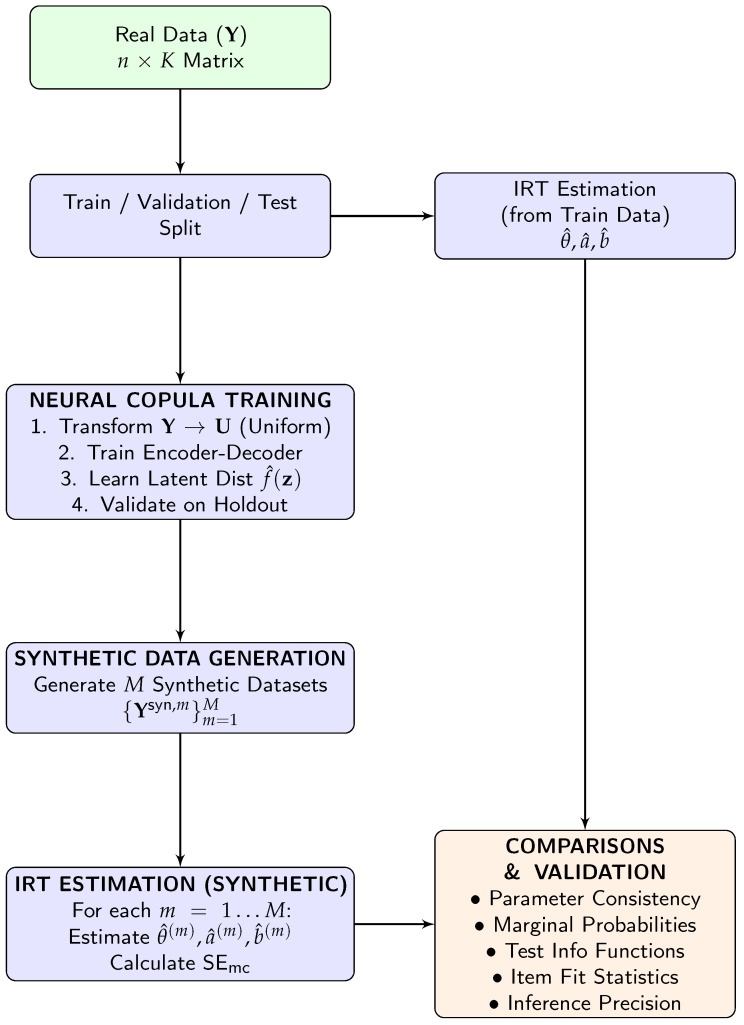
Flowchart of the Neural Network Copula analysis pipeline for synthetic data.

The analytical pipeline (Figure 1) employs the four-facet validation framework described in Section 2.3 to assess NNC synthetic data for IRT applications in assessment. After conducting a sensitivity analysis using 50 synthetic replications per configuration to establish the optimal latent dimensionality (*d*) and kernel density bandwidth (*h*), we structured the evaluation across the four validation facets. Table 1 summarizes the specific dataset allocations and random seed configurations utilized for each facet. To ensure reproducibility, the complete implementation code and sampled dataset are publicly available at https://github.com/Ju-youngJung/Neural-Network-Copula-Synthetic-Data.git (accessed on 14 April 2026).

The data-level consistency and psychometric property evaluations utilize a single primary synthetic dataset generated with a fixed seed of 42. Benchmark real parameters were estimated from the training data partition ($n_{\mathrm{train}}$) via MML estimation for the 2PL model using the girth 0.8.0 of the Python 3.11 package (Sanchez, 2021). At the data level, this single dataset supports the analysis of total score distributions, marginal probabilities, and inter-item correlations. At the psychometric level, this exact same dataset is reused to estimate IRT parameters (*a*, *b*), ICCs, TIF, CSEM, and item fit statistics. To distinguish generative model uncertainty from sampling variability (Robert & Casella, 2004), the statistical inference precision facet generates M=500 independent datasets using derived child seeds from the master random number generator.

Each dataset matches the training sample size ($n_{\mathrm{train}}=3244$). This volume ensures sufficient convergence of the MCSE estimator in Equation (20) (Koehler et al., 2009) while maintaining computational feasibility. IRT parameters $\left\{{\hat{a}}_{k}^{\left(m\right)},{\hat{b}}_{k}^{\left(m\right)}\right\}$ are re-estimated for each replication to compute the MCSE per item, which is then compared against the asymptotic standard error via Equation (21). A ratio of ${\mathrm{SE}}_{\mathrm{mc}}/{\mathrm{SE}}_{\mathrm{asym}}$ near 1.0 indicates that the synthetic data replicate the inferential precision of the original data, with ratios above 1.0 indicating conservative uncertainty and ratios below 1.0 indicating potential over-smoothing. The algorithmic stability facet restarts the entire pipeline from scratch across five distinct seeds (101, 202, 303, 404, 505). For each seed, the NNC is retrained from a random initialization, and one synthetic dataset is generated to confirm that parameter consistency metrics remain stable across optimization trajectories. The mean and standard deviation of $r_{a}$, $r_{b}$, and ${\mathrm{MAD}}_{\mathrm{marginal}}$ are reported to quantify sensitivity to random initialization.

### 3.2. Data Source and Partitioning

The empirical analysis utilized item response data from a large-scale high school mathematics assessment measuring numerical reasoning ability, a construct closely related to fluid intelligence and general cognitive ability. The dataset comprised binary responses (0 for incorrect, 1 for correct) from n=5069 examinees across K=20 items, yielding a response matrix $Y\in {\left\{0,1\right\}}^{n\times K}$ measuring a single latent ability construct consistent with the unidimensional 2PL model. Due to privacy protocols governing the operational records, the associated GitHub, repository provides a randomly sampled dataset of 3000 examinees for public replication. To separate optimization from evaluation while preserving the total score distribution, we partitioned the full operational data into three mutually exclusive subsets via stratified random sampling by score decile. These subsets included a training set (64%, $n_{\mathrm{train}}=3244$) to fit the NNC model and estimate benchmark IRT parameters, a validation set (16%, $n_{\mathrm{val}}=811$) for hyperparameter tuning and early stopping, and a test set (20%, $n_{\mathrm{test}}=1014$) for out-of-sample verification. Primary psychometric comparisons evaluated synthetic data generated from the model trained on $n_{\mathrm{train}}$ directly against this identical training partition. Estimating both the benchmark IRT parameters and the NNC model from the exact same data ensures a fair evaluation of generative fidelity independent of train and test distributional shifts.

## 4. Results

**Figure 2:**
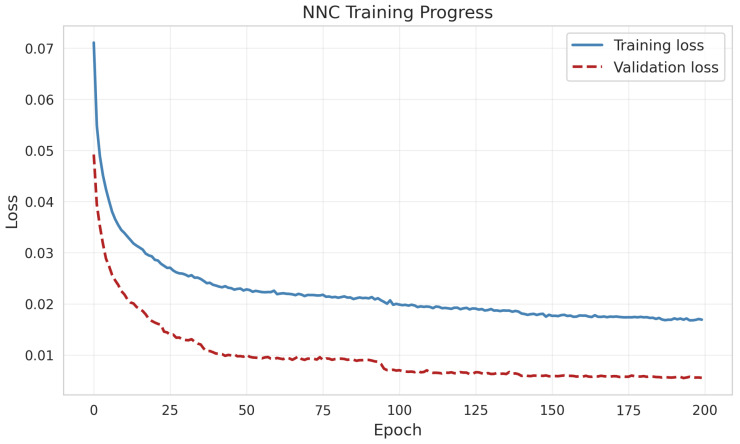
Neural Network Copula training progress. The plot shows the minimization of training loss (blue, solid) and validation loss (red, dashed) over 200 epochs.

All computations utilized PyTorch 1.12 (Paszke et al., 2019) within an environment featuring 32 CPU cores and a dedicated NVIDIA L40S GPU with CUDA acceleration. Training the model on the full dataset ($n_{\mathrm{train}}=3244$, K=20) to convergence required 679.4 s (approximately 11.3 min). Once trained, generating the primary synthetic dataset took only 0.02 s. Completing the M=500 Monte Carlo replications for standard error estimation required 367.4 s (approximately 6.1 min). Figure 2 illustrates the convergence trajectory of the composite loss function during training. Both the training and validation loss decreased rapidly within the first 50 epochs and stabilized thereafter, reaching a clear plateau by approximately epoch 150. The consistent alignment between the two curves indicates that the NNC successfully learned the underlying dependence structure of the response data without overfitting or optimization instability.

### 4.1. Sensitivity Analysis

We selected d=20 latent dimensions to optimize the balance between reconstruction fidelity and parsimony (Table 2). The candidate set d∈{10,20,30,50} was chosen to span a range from under-parameterized to over-parameterized representations relative to the number of observed items (K=20). Specifically, d=10 represents a compressed latent space (approximately half of *K*), which tests the risk of information loss, whereas d=30 and d=50 exceed the observed dimensionality and allow evaluation of potential overfitting and redundancy in the latent representation. This range ensures that the selected dimensionality is not driven by an arbitrary choice but reflects a principled exploration of model capacity.

Evaluating a validation set across d∈{10,20,30,50} using M=50 synthetic replications per configuration revealed that d=10 caused over-compression, severely degrading ${\mathrm{MAD}}_{\mathrm{marginal}}$ (0.032) and RMSR (0.065) despite yielding the highest $r_{a}$ (0.980) and a relatively lower $r_{b}$ (0.988) compared to higher dimensions. Among higher dimensions, d=20 achieved the highest $r_{a}$ (0.942) with $r_{b}=0.996$, while maintaining an excellent ${\mathrm{MAD}}_{\mathrm{marginal}}$ (0.015) and RMSR (0.030) comparable to d=30. Notably, $r_{b}$ remained stable at 0.996 across all d≥20, indicating that difficulty parameter recovery is robust to latent dimension choice within this range, whereas $r_{a}$ showed greater sensitivity. This configuration naturally bounds model capacity to one coordinate per item (K=20), relying on dropout and batch normalization to prevent trivial identity mapping.

For KDE, we employed a fixed bandwidth of h=0.2 to preserve meaningful tail densities for extreme scores. The candidate set h∈{0.1,0.2,0.3,0.5} was selected to represent a spectrum of smoothing levels, ranging from low smoothing (capturing fine-grained local structure) to high smoothing (emphasizing global density structure). In particular, h=0.1 allows evaluation of potential high-variance, under-smoothed estimates, whereas h=0.5 tests the impact of aggressive smoothing that may obscure inter-item dependencies. Intermediate values (*h* = 0.2, 0.3) provide practical trade-offs commonly recommended in the kernel density estimation literature.

Sensitivity evaluations across h∈{0.1,0.2,0.3,0.5}, also averaged over M=50 replications, confirmed this choice. Across all bandwidth values, $r_{b}$ remained highly stable (0.995–0.996), while $r_{a}$ ranged from 0.949 to 0.973 and RMSR varied more substantially. While overall performance remained stable for values up to h=0.3, the widest bandwidth (h=0.5) over-smoothed the inter-item dependence structure, resulting in a substantially higher RMSR (0.070). Ultimately, h=0.2 yielded the optimal balance with the lowest RMSR (0.025), $r_{b}=0.996$, and a ${\mathrm{MAD}}_{\mathrm{marginal}}$ of 0.016.

### 4.2. Data-Level Fidelity

#### 4.2.1. Total Score Distribution

**Figure 3:**
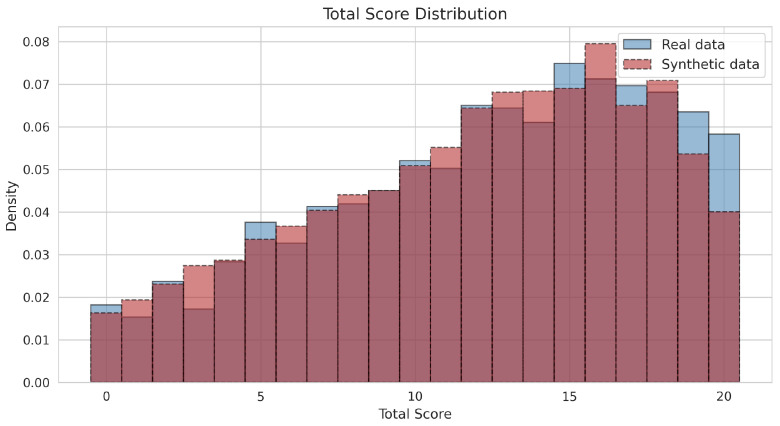
Comparison of observed total score distributions for real (blue, solid) and synthetic (red, dashed) data.

Generative quality at the respondent level was evaluated by comparing the distribution of observed total scores. As illustrated in Figure 3, the NNC accurately reproduced the distributional shape, central tendency, and dispersion of the empirical scores. The synthetic data closely matched the real data in terms of mean total score ($M_{\mathrm{syn}}=11.934$ versus $M_{\mathrm{real}}=12.233$) and standard deviation ($SD_{\mathrm{syn}}=5.293$ versus $SD_{\mathrm{real}}=5.344$). The synthetic distribution (red, dashed) closely overlaps with the real distribution (blue, solid), successfully capturing the distinct negative skewness (Skewness_syn_ = −0.441 versus Skewness_real_ = −0.459) and kurtosis (Kurtosis_syn_ = −0.764 versus Kurtosis_real_ = −0.735), as well as density peaks in the upper score range (scores 15 to 19). However, the synthetic data exhibited a slight underrepresentation of the maximum possible score (20), only partially capturing the frequency of perfect scores in the empirical data. This boundary effect is consistent with the known tendency of fixed-bandwidth KDE to underestimate density at distributional boundaries (Jones, 1993).

#### 4.2.2. Marginal Probabilities

**Figure 4:**
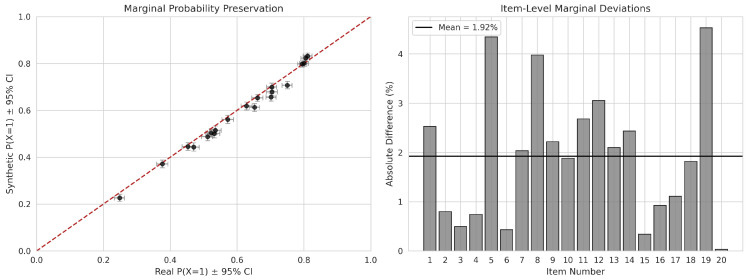
Consistency of marginal probabilities. The **left** panel illustrates the preservation of item difficulties relative to the identity line (red, dashed). The **right** panel quantifies absolute deviations per item with bootstrap 95% confidence intervals (whiskers) derived from 1000 resamples of the training data.

Item-level difficulty hierarchies were evaluated by contrasting the proportion of correct responses ($P(Y_{k}=1)$). Figure 4 (left panel) presents a scatter plot of these probabilities, revealing a strong linear relationship where points lie almost precisely on the identity line (r=0.981). This indicates that the NNC maintained the rank-order difficulty of items, successfully distinguishing between easy items (Item 13, $P_{\mathrm{real}}=0.811$) and difficult items (Item 09, $P_{\mathrm{real}}=0.248$). As detailed in Table 3 and visualized in Figure 4 (right panel), the ${\mathrm{MAD}}_{\mathrm{marginal}}$ across all 20 items was 0.019, with a maximum absolute difference of 0.045 for Item 19. The mean relative error of 3.398% demonstrates that univariate statistical features were preserved with high consistency. The right panel also displays bootstrap 95% confidence intervals computed from 1000 resamples of the training data; synthetic marginals fall within these intervals for all but one item, confirming that observed deviations are within expected sampling variability.

#### 4.2.3. Pairwise Associations

**Figure 5:**
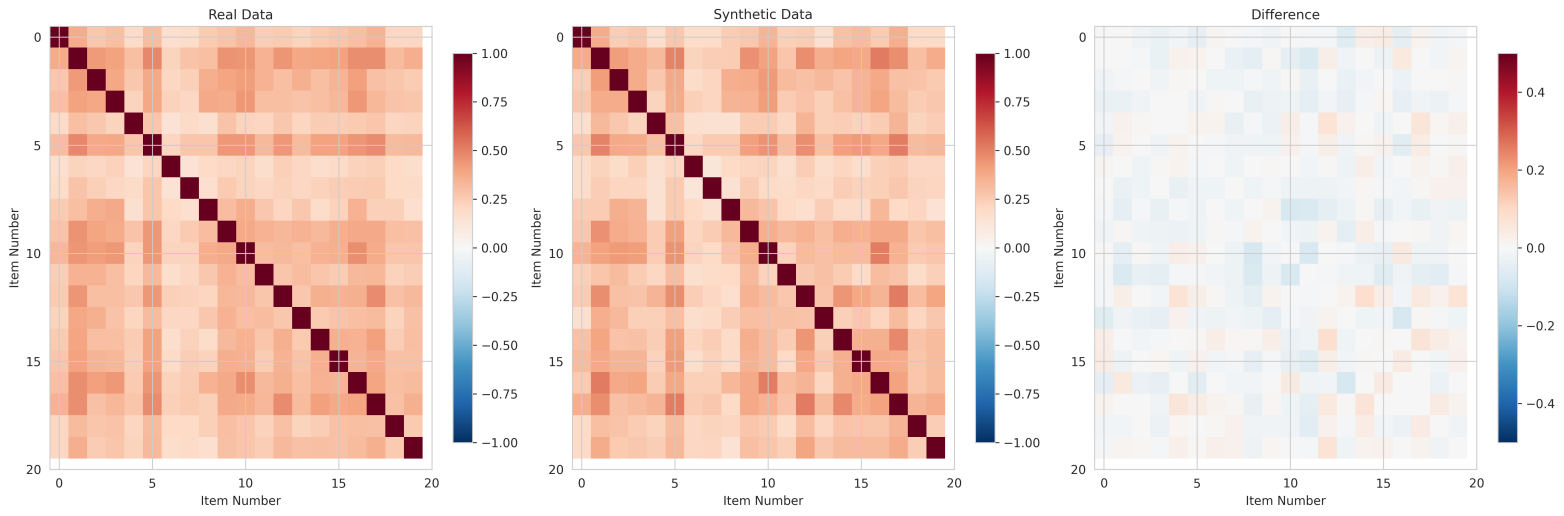
Inter-item correlation heatmaps for real data (**left)**, synthetic data (**middle**), and their difference (**right**). Each panel uses an identical colour scale. The colour scale for the difference matrix (**right**) ranges from −0.4 (blue) to +0.4 (red).

The preservation of higher-order dependencies was assessed by examining the inter-item correlation structures. Figure 5 presents the correlation matrices for the real data (left) and synthetic data (middle), alongside the difference matrix (right). Visual inspection confirms that the synthetic data accurately replicated the block-diagonal structure and intensity of the empirical correlations. The difference heatmap is predominantly neutral, indicating residuals close to zero and devoid of systematic bias. Quantitatively, this structural fidelity was confirmed by a low RMSR (0.032; values below 0.05 are generally considered indicative of good fit (Hu & Bentler, 1999)), validating that the NNC effectively preserved the local dependence structure required for accurate psychometric dimensionality assessment.

### 4.3. Psychometric Parameter Recovery

#### 4.3.1. IRT Parameter Consistency

**Figure 6:**
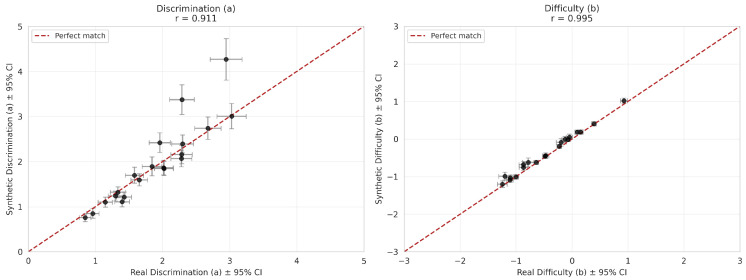
Scatter plots of IRT parameters for the representative synthetic dataset versus real data. The left panel shows discrimination (**a**) parameters and the right panel shows difficulty (**b**) parameters. The red dashed line indicates a perfect match.

**Figure 7:**
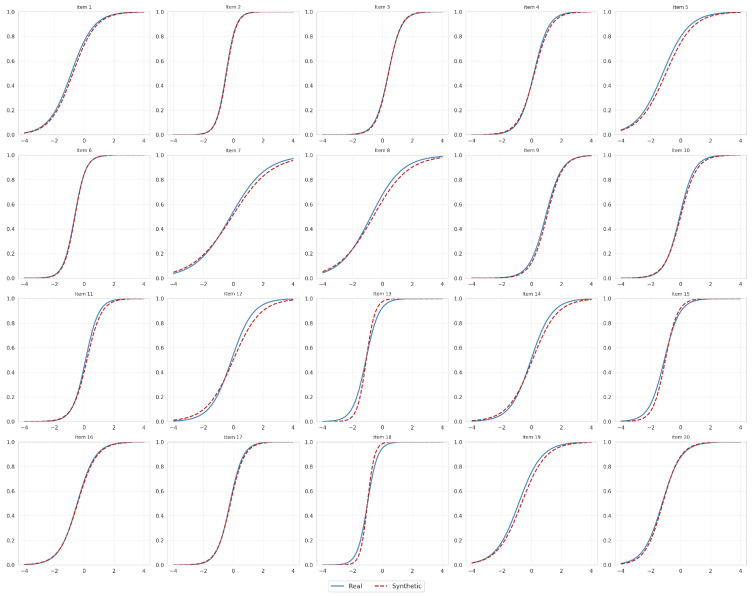
Item Characteristic Curves (ICCs) for real (blue, solid) and synthetic (red, dashed) data across all 20 items. Each panel plots the probability of a correct response $P(Y_{ik}=1|\theta )$ as a function of latent ability θ∈[−4,4].

The psychometric validity of the synthetic data was evaluated by examining the consistency of IRT parameter estimates and measurement precision. As illustrated in Figure 6, the discrimination parameters (*a*) showed strong alignment (r=0.911), although with slightly higher variability (${\mathrm{MAD}}_{\psi }=0.238$, ${\mathrm{RMSD}}_{\psi }=0.414$), primarily driven by items with high discrimination values (e.g., Items 13 and 18), as discussed further below. The difficulty parameters (*b*) exhibited exceptional consistency (r=0.995, ${\mathrm{MAD}}_{\psi }=0.075$, ${\mathrm{RMSD}}_{\psi }=0.095$). Despite the outliers in discrimination, the ICCs presented in Figure 7 demonstrate that the probability functions of the synthetic items closely track the empirical functions across the ability scale.

A notable pattern warrants specific discussion. Items 13 and 18, which possess high real discrimination values ($a_{13}=2.289$, $a_{18}=2.947$), exhibited the largest positive bias in the synthetic data ($a_{13}^{\mathrm{syn}}=3.374$, $a_{18}^{\mathrm{syn}}=4.266$). This amplification is systematic rather than random: the corresponding MCSEs were substantially inflated (${\mathrm{SE}}_{\mathrm{mc}}=0.169$ and 0.234, respectively, versus asymptotic estimates of 0.094 and 0.121; Table 4), and across all M=500 replications the mean synthetic discrimination for these items consistently exceeded the real values (Table 5). The ICCs for Items 13 and 18 in Figure 7 confirm that synthetic response functions remain plausible across the ability continuum, with the primary consequence being a sharp concentration of information near θ=−1.0. This location reflects the real data: the empirical difficulty parameters ($b_{13}=-1.109$, $b_{18}=-1.001$) indicate that the original assessment purposefully anchored highly discriminating items at this lower ability threshold to distinguish baseline proficiency.

**Figure 8:**
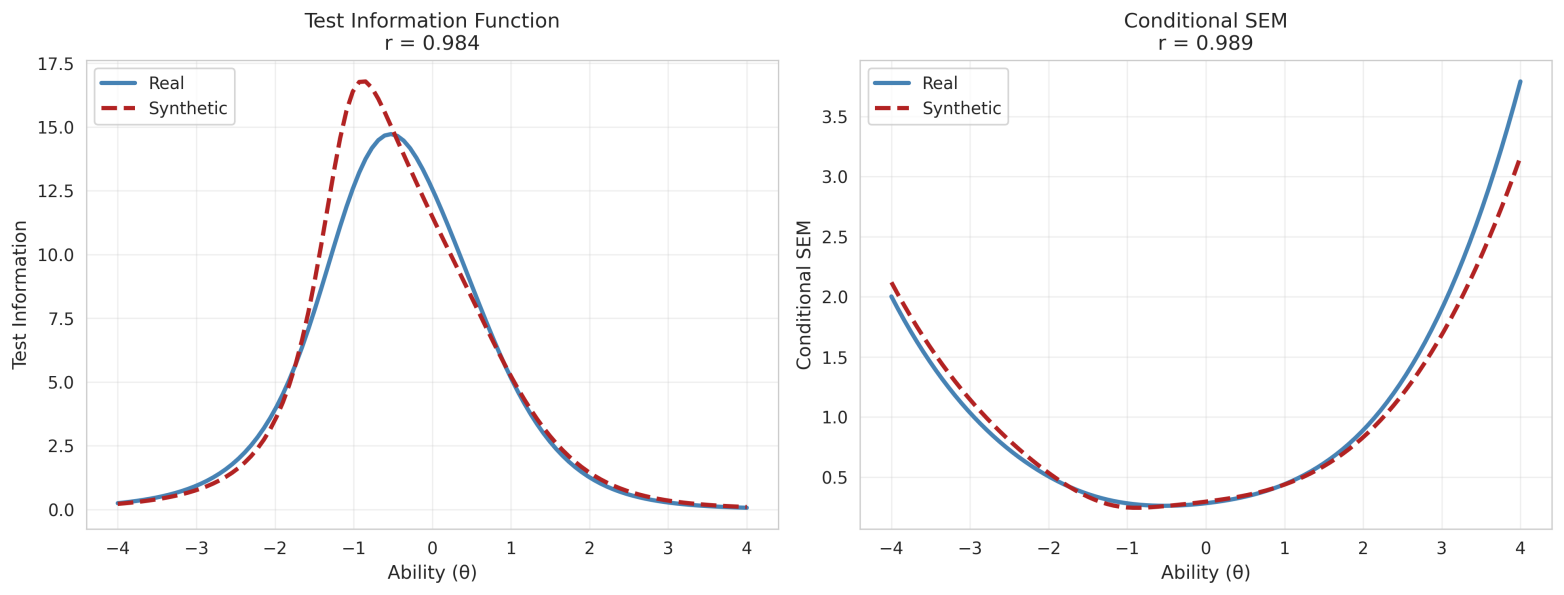
Test Information Function (TIF; **left** panel) and Conditional Standard Errors of Measurement (CSEM; **right** panel) for real (blue, solid) and synthetic (red, dashed) data. Curves are evaluated over a uniform grid of 100 points, θ∈[−4,4].

To evaluate measurement precision across the latent continuum, we examined the TIF and CSEM. As shown in Figure 8, the synthetic TIF curve follows the general trajectory of the real data, with both curves peaking in the lower ability range (θ≈−1.0) and declining symmetrically toward the extremes of the scale. Although slightly higher peak information appeared around θ=−1.0 due to the high-discrimination amplification noted above, the overall profiles are highly consistent, yielding a TIF vector correlation of r=0.984. The corresponding CSEM profiles mirror this pattern inversely, as measurement error is minimized precisely where information is maximized. The synthetic CSEM curve closely tracked the real curve across the full ability range, with near-identical minimum error values near θ=−1.0 and equivalent error inflation at the extremes (θ<−3 and θ>2), yielding a CSEM vector correlation of r=0.989. Together, these results indicate that the synthetic data preserves the measurement precision profile of the original instrument, supporting its use in applications requiring standard error estimation across the ability continuum.

Systematic bias was evaluated by comparing real item parameters against the mean synthetic estimates across all M=500 replications (Table 5). For discrimination parameters (*a*), two opposing tendencies emerged. For the majority of items with moderate discrimination (Items 4, 11, 12, and 16), a slight attenuation bias was observed, with mean synthetic estimates under-predicting empirical values by approximately 0.069 to 0.172. This attenuation is consistent with the regularizing effect of dropout (p=0.2) and batch normalization in the encoder (Srivastava et al., 2014). Conversely, significant positive bias appeared for Item 18 (+1.075), Item 13 (+0.889), and Item 15 (+0.416), reflecting the KDE-induced amplification mechanism described above. For difficulty parameters (*b*), absolute differences were consistently low (mean absolute bias = 0.051), with the largest deviation for Item 5 (0.229).

#### 4.3.2. Item Fit Statistics

**Figure 9:**
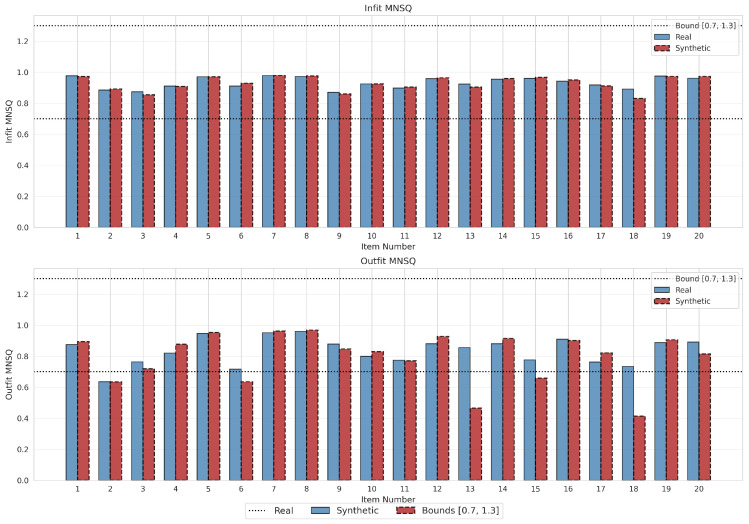
Comparison of Infit (upper panel) and Outfit (lower panel) Mean Square statistics for real data (blue, solid) and synthetic data (red, dashed). Horizontal dotted lines indicate the acceptable fit range [0.7, 1.3].

Infit statistics demonstrated remarkable stability across all 20 items, with the average absolute deviation between real and synthetic values being negligible (<0.01), and the majority of items well within the acceptable range [0.7, 1.3] for both datasets (Wright & Stone, 1979). Outfit statistics were largely preserved, with most items showing differences of less than 0.05. However, two items revealed localized under-dispersion: Item 13 showed a reduction from 0.856 (real) to 0.466 (synthetic), and Item 18 from 0.733 to 0.413, both falling below the 0.5 threshold for severe under-dispersion and flagged accordingly in Table 6. Item 15 showed a moderate reduction (0.776 → 0.659) but remained above the critical threshold. These patterns are consistent with the discrimination amplification mechanism described above: high-discrimination items generated response patterns that were too deterministic, suppressing the stochastic noise typically found in empirical outlier responses. Results are visualized in Figure 9.

### 4.4. Inferential Precision

The synthetic data generally produced slightly larger standard errors than the real data, indicating conservative estimation of precision. On average, MCSEs exceeded ASEs for both discrimination (0.095 versus 0.074) and difficulty (0.037 versus 0.031) parameters, yielding mean MCSE/ASE ratios of 1.286 and 1.179, respectively. This conservative pattern was non-uniform across items. Items with low-to-moderate discrimination showed near-exact correspondence (e.g., Item 7: ${\mathrm{SE}}_{\mathrm{asym}}=0.044$, ${\mathrm{SE}}_{\mathrm{mc}}=0.045$, ratio = 1.005), while high-discrimination items exhibited substantial inflation (Item 13: ratio = 1.791; Item 18: ratio = 1.936), consistent with the amplification mechanism described in the IRT Parameter Consistency subsection above. Full item-level SE comparisons are reported in Table 4.

### 4.5. Stability Across Initializations

Robustness was assessed by repeating the full analysis pipeline with five distinct random seeds {101, 202, 303, 404, 505} to evaluate sensitivity to initialization and stochastic optimization paths. As summarized in Table 7, discrimination parameters (*a*) showed strong but slightly more variable consistency, ranging from r=0.929 (Seed 101) to r=0.954 (Seed 303), averaging 0.944 (SD=0.010). Difficulty parameters (*b*) exhibited near-perfect convergence, with correlations ranging from r=0.992 (Seed 505) to r=0.996 (Seeds 101, 303, and 404), yielding a mean of 0.995. The ${\mathrm{MAD}}_{\mathrm{marginal}}$ ranged from 0.014 (Seeds 202 and 404) to 0.019 (Seed 101), with a mean of 0.016 (SD=0.002). These results confirm that the NNC reliably converges to a stable structural representation of the assessment data regardless of random initialization state.

## 5. Discussion

This study introduced and validated an NNC framework for generating synthetic item response data in assessment while preserving psychometric integrity. By decoupling marginal probabilities from the dependence structure, the NNC circumvents the rigid distributional assumptions of traditional parametric IRT simulation and addresses structural gaps in existing deep generative alternatives such as GANs and VAEs, which lack explicit mechanisms for preserving marginal item properties.

### 5.1. Synthesis of Findings

The four-facet validation demonstrated high fidelity throughout. At the data-level, total score distributions were accurately reproduced ($M_{\mathrm{real}}=12.233$ versus $M_{\mathrm{syn}}=11.934$), marginal probabilities were well preserved (${\mathrm{MAD}}_{\mathrm{marginal}}=0.019$, r=0.981), and inter-item correlation structure was maintained (RMSR=0.032). This confirms that the copula encoding captured higher-order dependencies essential for dimensionality assessment in psychometric measurement. Psychometric parameter recovery was strong, with difficulty parameters showing exceptional consistency (r=0.995, ${\mathrm{MAD}}_{b}=0.075$) and TIF and CSEM vector correlations remaining high ($r_{\mathrm{TIF}}=0.984$, $r_{\mathrm{CSEM}}=0.989$). This indicates that the synthetic data retains sufficient measurement precision for downstream applications such as test assembly and equating. The Monte Carlo analysis (M=500) yielded slightly conservative standard error estimates (mean MCSE/ASE ratios of 1.286 for discrimination and 1.179 for difficulty), suggesting that the NNC effectively functions as a generative bootstrapping mechanism that captures model misspecification uncertainty absent from analytical ASEs (Efron & Tibshirani, 1994). Stability across initializations was confirmed across five independent retraining runs (${\bar{r}}_{a}=0.944$, SD=0.010; ${\bar{r}}_{b}=0.995$, SD=0.002), demonstrating robustness to random initialization.

### 5.2. High-Discrimination Item Behavior

A systematic amplification of discrimination parameters was observed for the highest-discriminating items alongside attenuation for items of moderate discrimination. The attenuation reflects regularization via dropout and batch normalization, which produce a smoothed representation of the dependence structure (Ioffe & Szegedy, 2015; Srivastava et al., 2014). This shrinkage is generally benign, as Infit statistics remained stable across all items. The amplification for Items 13 and 18 (mean synthetic biases of +0.889 and +1.075 respectively across M=500 replications) is more consequential. High-discrimination items produce sharply clustered response patterns that create high-density regions in the latent space; the fixed-bandwidth KDE (h=0.2) over-samples these clusters, generating synthetic latent codes that decode to more deterministic response probabilities, inflating discrimination estimates and suppressing Outfit statistics below the 0.5 severe under-dispersion threshold (Wright & Stone, 1979). Adaptive bandwidth selection (Silverman, 1986) or targeted regularization tuning represent the most tractable remedies; practitioners should consider down-weighting items flagged for severe under-dispersion in discrimination-sensitive applications such as cognitive ability test assembly.

### 5.3. Limitations and Future Directions

Several limitations warrant consideration. First, the fixed-bandwidth KDE produced a slight underrepresentation of the maximum total score (20), consistent with known boundary bias (Jones, 1993). Boundary-corrected kernel methods or adaptive bandwidth selection would address this issue. Second, the framework is currently scoped to dichotomous responses within a unidimensional 2PL model. Extending this to polytomous formats like the Graded Response Model (Samejima, 1969) and multidimensional IRT structures (Reckase, 2009) represents a natural next step. These extensions are particularly relevant for cognitive batteries involving mixed response formats, though they introduce additional decoding complexity and heighten the curse of dimensionality in KDE (Scott, 2015). Third, transitioning to large-scale operational assessment settings introduces scalability considerations. Expanding to massive sample sizes inherently benefits deep learning models by providing the volume necessary to stabilize complex autoencoder training and accurately map the latent space. This transition requires implementing computationally efficient density estimation techniques such as normalizing flows (D. Rezende & Mohamed, 2015). Finally, using empirical data precludes evaluation against known ground-truth parameters. Future simulation studies with fixed population values would clarify the bias-variance trade-off of the NNC estimator relative to standard MML approaches.

## 6. Conclusions

This study demonstrated that the NNC framework offers a robust and data-driven alternative to traditional parametric simulation and existing deep generative models for synthetic item response data generation. The four-facet validation confirmed high fidelity across data-level consistency, psychometric parameter recovery, test information preservation, and inferential precision. Furthermore, the MCSE/ASE ratios establish the NNC as a principled generative bootstrapping mechanism. The primary limitation identified was the amplification of discrimination for highly discriminating items. This artifact is attributable to the fixed-bandwidth KDE and represents a tractable target for future refinement that does not undermine the overall validity of the framework. As the volume and complexity of educational and psychological assessment data continue to expand, flexible generative architectures of this kind will prove increasingly vital for securely sharing data, enabling reproducible research, and benchmarking emerging psychometric methodologies.

## Figures and Tables

**Table 1:** Summary of synthetic dataset allocation and seed configuration across the four validation facets.

Validation Facet	Datasets	Seeds
Data-Level Consistency	1	42
Psychometric Properties	1	42
Statistical Inference Precision	500	Derived from master RNG
Algorithmic Stability	5	101, 202, 303, 404, 505

**Table 2:** Sensitivity of validation metrics to latent dimension (*d*) and kernel density estimation bandwidth (*h*).

Parameter	Value	$r_{a}$	$r_{b}$	${\mathrm{MAD}}_{\mathrm{marginal}}$	RMSR
Latent dim (*d*)	10	0.980	0.988	0.032	0.065
	**20**	**0.942**	**0.996**	**0.015**	**0.030**
	30	0.903	0.996	0.016	0.027
	50	0.910	0.996	0.016	0.028
Bandwidth (*h*)	0.1	0.949	0.996	0.015	0.030
	**0.2**	**0.954**	**0.996**	**0.016**	**0.025**
	0.3	0.956	0.995	0.017	0.028
	0.5	0.973	0.995	0.019	0.070

Note. Bold rows indicate the selected configurations. $r_{a}$ and $r_{b}$ = Pearson correlations between real and synthetic discrimination and difficulty parameters, respectively. ${\mathrm{MAD}}_{\mathrm{marginal}}$ = mean absolute deviation of marginal probabilities. RMSR = root mean square residual of inter-item correlations. Each cell is the mean across M=50 synthetic replications per configuration.

**Table 3:** Comparison of real and synthetic marginal probabilities ($P(Y_{k}=1)$).

Item	$P_{\mathrm{Real}}$	$P_{\mathrm{Syn}}$	Absolute Difference	Relative Difference (%)
01	0.704	0.679	0.025	3.589
02	0.661	0.653	0.008	1.212
03	0.376	0.371	0.005	1.310
04	0.453	0.445	0.007	1.634
05	0.750	0.707	0.043	5.793
06	0.704	0.699	0.004	0.613
07	0.535	0.515	0.020	3.802
08	0.652	0.613	0.040	6.096
09	0.248	0.226	0.022	8.933
10	0.523	0.504	0.019	3.599
11	0.470	0.444	0.027	5.701
12	0.531	0.501	0.031	5.742
13	0.811	0.832	0.021	2.585
14	0.512	0.487	0.024	4.759
15	0.795	0.798	0.003	0.427
16	0.628	0.619	0.009	1.473
17	0.572	0.561	0.011	1.941
18	0.805	0.823	0.018	2.260
19	0.702	0.656	0.045	6.459
20	0.800	0.801	0.000	0.039
Mean			0.019	3.398

Note. Syn = Synthetic. Relative Difference = $|P_{\mathrm{real}}-P_{\mathrm{syn}}|/P_{\mathrm{real}}\times 100$.

**Table 4:** Comparison of real and synthetic item parameters and standard errors.

Item	Discrimination (*a*)		SE (*a*)		Difficulty (*b*)		SE (*b*)	
	Real	Syn	Asym	MC	Real	Syn	Asym	MC
1	1.334	1.319	0.057	0.062	−0.866	−0.754	0.040	0.042
2	3.028	3.006	0.111	0.143	−0.481	−0.456	0.019	0.027
3	2.299	2.391	0.082	0.102	0.394	0.405	0.021	0.029
4	2.022	1.847	0.072	0.078	0.155	0.187	0.022	0.030
5	1.147	1.101	0.055	0.057	−1.200	−0.990	0.055	0.053
6	2.674	2.740	0.100	0.127	−0.639	−0.620	0.021	0.029
7	0.848	0.758	0.044	0.045	−0.192	−0.088	0.045	0.052
8	0.960	0.850	0.048	0.053	−0.780	−0.622	0.051	0.060
9	1.843	1.894	0.074	0.106	0.929	1.017	0.031	0.036
10	2.016	1.864	0.072	0.082	−0.074	−0.013	0.022	0.028
11	2.280	2.068	0.080	0.093	0.093	0.184	0.020	0.028
12	1.398	1.108	0.056	0.057	−0.123	−0.004	0.029	0.035
13	2.289	3.374	0.094	0.169	−1.109	−1.087	0.031	0.033
14	1.428	1.213	0.056	0.057	−0.045	0.054	0.029	0.038
15	1.958	2.417	0.081	0.112	−1.100	−1.032	0.034	0.038
16	1.653	1.595	0.063	0.069	−0.468	−0.441	0.028	0.036
17	2.283	2.157	0.081	0.104	−0.227	−0.196	0.021	0.028
18	2.947	4.266	0.121	0.234	−1.001	−1.005	0.024	0.032
19	1.301	1.236	0.056	0.056	−0.866	−0.678	0.041	0.042
20	1.582	1.697	0.069	0.090	−1.243	−1.202	0.044	0.045

Note. Syn = representative synthetic dataset (seed = 42). Asym = asymptotic standard error from the Fisher information matrix of the real data. MC =Monte Carlo from *M* = 500 independent replications. SE = standard error.

**Table 5:** Comparison of real item parameters and mean synthetic estimates across M=500 replications.

Item	Discrimination (*a*)			Difficulty (*b*)		
	Real	Mean Synthetic	Difference	Real	Mean Synthetic	Difference
1	1.334	1.384	0.050	−0.866	−0.736	0.130
2	3.028	3.075	0.047	−0.481	−0.464	0.017
3	2.299	2.307	0.008	0.394	0.376	−0.018
4	2.022	1.850	−0.172	0.155	0.160	0.005
5	1.147	1.163	0.016	−1.200	−0.971	0.229
6	2.674	2.767	0.093	−0.639	−0.622	0.017
7	0.848	0.747	−0.101	−0.192	−0.211	−0.019
8	0.960	0.869	−0.091	−0.780	−0.693	0.087
9	1.843	2.121	0.278	0.929	0.908	−0.021
10	2.016	1.940	−0.076	−0.074	−0.028	0.046
11	2.280	2.211	−0.069	0.093	0.151	0.058
12	1.398	1.289	−0.109	−0.123	−0.091	0.032
13	2.289	3.178	0.889	−1.109	−1.074	0.035
14	1.428	1.261	−0.167	−0.045	−0.008	0.037
15	1.958	2.374	0.416	−1.100	−1.089	0.011
16	1.653	1.545	−0.108	−0.468	−0.456	0.012
17	2.283	2.347	0.064	−0.227	−0.232	−0.005
18	2.947	4.022	1.075	−1.001	−1.001	0.000
19	1.301	1.213	−0.088	−0.866	−0.709	0.157
20	1.582	1.876	0.294	−1.243	−1.162	0.081

Note. Mean Synthetic = mean synthetic estimate across M=500 replications. Positive difference values indicate upward bias; negative values indicate attenuation bias.

**Table 6:** Comparison of Item Infit and Outfit Mean Square statistics for real and synthetic data.

Item	Infit Mean Square		Outfit Mean Square	
	Real	Synthetic	Real	Synthetic
1	0.976	0.972	0.875	0.895
2	0.885	0.893	0.635	0.635
3	0.874	0.855	0.763	0.720
4	0.911	0.908	0.820	0.877
5	0.970	0.971	0.946	0.953
6	0.911	0.929	0.716	0.636
7	0.978	0.979	0.951	0.963
8	0.972	0.975	0.960	0.969
9	0.870	0.860	0.879	0.848
10	0.924	0.925	0.800	0.830
11	0.898	0.905	0.774	0.771
12	0.959	0.964	0.881	0.928
13	0.923	0.904	0.856	0.466 ^†^
14	0.955	0.959	0.880	0.915
15	0.960	0.966	0.776	0.659
16	0.942	0.950	0.909	0.902
17	0.917	0.912	0.763	0.821
18	0.891	0.831	0.733	0.413 ^†^
19	0.976	0.974	0.887	0.906
20	0.962	0.974	0.892	0.815

Note. Infit Mean Square = information-weighted mean square residual statistic. Outfit Mean Square = unweighted mean square residual statistic. Synthetic values are derived from a single representative synthetic dataset (seed = 42). Acceptable fit range [0.7, 1.3]. ^†^ Outfit Mean Square < 0.5: severe under-dispersion in synthetic data.

**Table 7:** Stability across initializations: Results across five independent random seeds.

Seed	$r_{a}$	$r_{b}$	${\mathrm{MAD}}_{\mathrm{marginal}}$
101	0.929	0.996	0.019
202	0.943	0.995	0.014
303	0.954	0.996	0.015
404	0.951	0.996	0.014
505	0.943	0.992	0.017
Mean	0.944	0.995	0.016
SD	0.010	0.002	0.002

Note. $r_{a}$ and $r_{b}$ are Pearson correlations between real and synthetic discrimination and difficulty parameters, respectively. ${\mathrm{MAD}}_{\mathrm{marginal}}$ = mean absolute deviation of marginal item probabilities. SD = standard deviation.

## Data Availability

The complete implementation code and a sampled dataset (provided due to privacy protocols) for public replication are openly available at https://github.com/Ju-youngJung/Neural-Network-Copula-Synthetic-Data.git (accessed on 14 April 2026).

## Abbreviations

The following abbreviations are used in this manuscript:

2PL	Two-Parameter Logistic
ASE	Asymptotic Standard Error
CSEM	Conditional Standard Error of Measurement
EAP	Expected A Posteriori
EM	Expectation-Maximization
GAN	Generative Adversarial Network
ICC	Item Characteristic Curve
IRT	Item Response Theory
KDE	Kernel Density Estimation
MAD	Mean Absolute Deviation
MCSE	Monte Carlo Standard Error
MML	Marginal Maximum Likelihood
NNC	Neural Network Copula
ReLU	Rectified Linear Unit
TIF	Test Information Function
VAE	Variational Autoencoder

## Appendix A. Neural Network Copula Algorithms

The following pseudocode details the complete pipeline for the NNC framework. Algorithm A1 outlines the model training procedure, including the transformation of binary responses to pseudo-uniform marginals and the optimization of the deep autoencoder. Algorithm A2 describes the synthetic data generation process, which utilizes KDE to sample from the learned latent space and the inverse probability integral transform to produce binary responses.

**Algorithm A1** Neural Network Copula Training Procedure
**Require:** Training data $Y_{\mathrm{train}}$ ($N_{\mathrm{train}}\times K$ binary matrix), Validation data $Y_{\mathrm{val}}$ ($N_{\mathrm{val}}\times K$) **Require:** Configuration {latent_dim=20,hidden_dims=[256,128,64],dropout=0.2,λ=0.1} **Ensure:** Trained model, marginal probabilities π, training history 1: **Step 1: Data Preprocessing** 2: Calculate empirical marginal probabilities: $\pi_{k}\leftarrow \frac{1}{N_{\mathrm{train}}}\sum_{i=1}^{N_{\mathrm{train}}}y_{i,k}^{\left(\mathrm{train}\right)}$ for all k=1,…,K 3: **for** i=1 **to** $N_{\mathrm{train}}$ **do** 4: **for** k=1 **to** *K* **do** 5: **if** $y_{i,k}^{\left(\mathrm{train}\right)}=1$ **then** 6: $u_{i,k}^{\left(\mathrm{train}\right)}\leftarrow \mathrm{Uniform}\left(1-\pi_{k},\;1\right)$ 7: **else** 8: $u_{i,k}^{\left(\mathrm{train}\right)}\leftarrow \mathrm{Uniform}\left(0,\;1-\pi_{k}\right)$ 9: **end if** 10: **end for** 11: **end for** 12: Apply the same transformation to $Y_{\mathrm{val}}\to U_{\mathrm{val}}$ using the training π {Training marginals used for validation to avoid data leakage} 13: **Step 2: Model Initialization** 14: Initialize **Encoder**: Input(K)→[256,128,64]→Latent(d=20) 15: Per-layer structure: Linear → ReLU → BatchNorm → Dropout(p=0.2) 16: Final encoder layer: Linear projection to d=20, *no activation* {Latent codes span full $R^{d}$} 17: Initialize **Decoder**: Latent(20)→[64,128,256]→Output(K) 18: Structure: mirror of Encoder, ending with Sigmoid activation {Constrains outputs to (0,1); distinct from the IRT logistic ICC} 19: Initialize Optimizer: Adam with η=0.001, $\beta_{1}=0.9$, $\beta_{2}=0.999$ 20: Initialize Scheduler: ReduceLROnPlateau (factor = 0.5, patience = 10) 21: **Step 3: Training Optimization Loop** 22: **for** epoch=1 **to** 200 **do** 23: **3.1 Training Phase** 24: Set model to train mode 25: **for** each mini-batch B in DataLoader($U_{\mathrm{train}}$, batch_size = 64) **do** 26: Z←Encoder(B) {Map to d=20 dimensional latent space} 27: $\hat{U}\leftarrow \mathrm{Decoder}\left(Z\right)$ {Reconstruct pseudo-uniform marginals} 28: $L_{\mathrm{recon}}\leftarrow \mathrm{MSE}\left(B,\;\hat{U}\right)$ 29: $L_{\mathrm{marg}}\leftarrow \mathrm{MSE}\;\left(\mathrm{ColMeans}(\hat{U}),\;0.5\right)$ {Prevents marginal collapse} 30: $L_{\mathrm{total}}\leftarrow L_{\mathrm{recon}}+\lambda \;L_{\mathrm{marg}}$ {λ=0.1} 31: Zero gradients → Backward pass → Update weights (Adam) 32: **end for** 33: **3.2 Validation Phase** 34: Set model to eval mode 35: Compute validation loss on $U_{\mathrm{val}}$ using same composite loss function 36: Update learning rate scheduler based on validation loss 37: **3.3 Early Stopping** 38: **if** validation loss improves **then** 39: Save current weights as *best model* 40: Reset patience counter ←0 41: **else** 42: Increment patience counter 43: **if** patience counter ≥20 **then** 44: **break** {Retain best model weights} 45: **end if** 46: **end if** 47: **end for** 48: **return** Best model state, π, training history

**Algorithm A2** Synthetic Data Generation via Neural Network Copula
**Require:** Trained model, training pseudo-uniform matrix $U_{\mathrm{train}}$, marginal probabilities π, number of synthetic samples $N_{\mathrm{syn}}$, random seed *s* **Ensure:** Synthetic binary response matrix $Y_{\mathrm{syn}}\in {\left\{0,1\right\}}^{N_{\mathrm{syn}}\times K}$ 1: **Step 1: Latent Space Characterization via KDE** 2: Set model to eval mode 3: Extract training latent codes: $Z_{\mathrm{train}}\leftarrow \mathrm{model}.\mathrm{encoder}\left(U_{\mathrm{train}}\right)$ {$Z_{\mathrm{train}}\in R^{N_{\mathrm{train}}\times d}$, d=20} 4: Fit Gaussian KDE $\hat{f}\left(z\right)$ on $Z_{\mathrm{train}}$: 5: Kernel: standard Gaussian 6: Bandwidth: h←0.2 {Selected via sensitivity analysis across h∈{0.1,0.2,0.3,0.5}; h=0.2 minimises RMSR (see Table 2)} 7: **Step 2: Latent Sampling** 8: Initialise child RNG from seed *s* {Ensures each replication is independently seeded and deterministic given the master seed} 9: Draw $N_{\mathrm{syn}}$ synthetic latent codes: $Z_{\mathrm{syn}}∼\hat{f}\left(z\right)$ {KDE sampling: draw a training code, add Gaussian noise $∼N(0,\;h^{2}I_{d})$} 10: **Step 3: Decode to Pseudo-Uniform Marginals** 11: $U_{\mathrm{syn}}\leftarrow \mathrm{model}.\mathrm{decoder}\left(Z_{\mathrm{syn}}\right)$ {Sigmoid output constrains $U_{ik}^{\mathrm{syn}}\in \left(0,1\right)$} 12: Clip: $U_{\mathrm{syn}}\leftarrow \mathrm{clip}\left(U_{\mathrm{syn}},\;0,\;1\right)$ 13: **Step 4: Inverse Probability Integral Transform** {Guarantees $E\left[Y_{ik}^{\mathrm{syn}}\right]=\pi_{k}$ by construction (Genest et al., 2009), preserving item difficulty. Item discrimination is preserved implicitly through the dependence structure encoded in $Z_{\mathrm{syn}}$.} 14: Initialize $Y_{\mathrm{syn}}\leftarrow 0_{N_{\mathrm{syn}}\times K}$ 15: **for** i=1 **to** $N_{\mathrm{syn}}$ **do** 16: **for** k=1 **to** *K* **do** 17: Threshold: $\tau_{k}\leftarrow 1-\pi_{k}$ 18: **if** $u_{i,k}^{\left(\mathrm{syn}\right)}>\tau_{k}$ **then** 19: $y_{i,k}^{\left(\mathrm{syn}\right)}\leftarrow 1$ 20: **else** 21: $y_{i,k}^{\left(\mathrm{syn}\right)}\leftarrow 0$ 22: **end if** 23: **end for** 24: **end for** 25: **return** $Y_{\mathrm{syn}}$
